# Widespread attenuating changes in brain connectivity associated with the general factor of psychopathology in 9- and 10-year olds

**DOI:** 10.1038/s41398-021-01708-w

**Published:** 2021-11-09

**Authors:** Chandra Sripada, Mike Angstadt, Aman Taxali, Daniel Kessler, Tristan Greathouse, Saige Rutherford, D. Angus Clark, Luke W. Hyde, Alex Weigard, Sarah J. Brislin, Brian Hicks, Mary Heitzeg

**Affiliations:** 1 Department of Psychiatry, University of Michigan, Ann Arbor, MI USA; 2 Department of Statistics, University of Michigan, Ann Arbor, MI USA; 3 Department of Psychology and Survey Research Center at the Institute for Social Research, University of Michigan, Ann Arbor, MI USA

**Keywords:** ADHD, Long-term memory

## Abstract

Convergent research identifies a general factor (“P factor”) that confers transdiagnostic risk for psychopathology. Large-scale networks are key organizational units of the human brain. However, studies of altered network connectivity patterns associated with the P factor are limited, especially in early adolescence when most mental disorders are first emerging. We studied 11,875 9- and 10-year olds from the Adolescent Brain and Cognitive Development (ABCD) study, of whom 6593 had high-quality resting-state scans. Network contingency analysis was used to identify altered interconnections associated with the P factor among 16 large-scale networks. These connectivity changes were then further characterized with quadrant analysis that quantified the directionality of P factor effects in relation to neurotypical patterns of positive versus negative connectivity across connections. The results showed that the P factor was associated with altered connectivity across 28 network cells (i.e., sets of connections linking pairs of networks); *p*_PERMUTATION_ values < 0.05 FDR-corrected for multiple comparisons. Higher P factor scores were associated with hypoconnectivity within default network and hyperconnectivity between default network and multiple control networks. Among connections within these 28 significant cells, the P factor was predominantly associated with “attenuating” effects (67%; *p*_PERMUTATION_ < 0.0002), i.e., reduced connectivity at neurotypically positive connections and increased connectivity at neurotypically negative connections. These results demonstrate that the general factor of psychopathology produces attenuating changes across multiple networks including default network, involved in spontaneous responses, and control networks involved in cognitive control. Moreover, they clarify mechanisms of transdiagnostic risk for psychopathology and invite further research into developmental causes of distributed attenuated connectivity.

## Introduction

Recent investigations into patterns of covariance across psychiatric symptoms consistently find a general factor of psychopathology, termed the “P factor”, which is associated with most prevalent psychiatric symptoms [1–4]. Concurrently, categorical diagnostic approaches that currently predominate encounter serious issues. Tellingly, one persistent problem has been excess overlap across disorders in symptoms [5], neural mechanisms [6], and genetic risk factors [7]—a problem that could be readily explained if a domain-general P factor drives co-occurrence of symptoms irrespective of diagnostic boundaries. Despite these compelling features of the P factor model, key gaps in knowledge remain, especially regarding the neural mechanisms that produce broad expression of diverse psychopathologies and the developmental pathways through which these mechanisms operate.

Network neuroscience [8–11] is well positioned to help fill in this gap in knowledge. The human brain is organized into a number of large-scale connectivity networks [12]. There is growing understanding of distinct information-processing functions implemented by these networks and by interacting network ensembles. Recent psychological models of the P factor emphasize heightened generation of impulses and reduced executive regulation [3, 13]. These findings raise the possibility that the P factor involves alterations in networks involved in the generation of spontaneous thought (default mode network, DMN) and bottom-up attention (ventral attention network, VAN), as well as networks involved in cognitive control (e.g., frontoparietal network, FPN, dorsal attention network, DAN; cingulo-opercular network, CO) [14]. Recent studies found altered functional-connectivity patterns associated with transdiagnostic dimensions [15–21], but findings have been mixed and await further clarification.

Network neuroscience can also illuminate the developmental pathways that lead to psychopathology. Brain networks undergo substantial maturation during adolescence [22–24]. Importantly, this is also the time that many serious mental disorders first emerge [5, 25, 26]. In neurotypical individuals, connectomic development is characterized by the emergence of complex patterns of variation across connections in positive connectivity, thought to represent information integration [27], and negative connectivity, thought to represent information segregation and/or inhibitory relationships [28], with the balance between integration and segregation potentially related to cognitive control [29] and processing efficiency [30]. However, though the P factor has been conceptualized as impacting neurodevelopment [3], the role of the P factor in modulating these complex patterns of positive versus negative connectivity, for example by attenuating neurotypical patterns of variation across connections, awaits detailed investigation.

The current study examines functional- connectivity patterns across the whole connectome associated with the P factor in a sample of 11,875 9- and 10-year olds in the Adolescent Brain and Cognitive Development (ABCD) national consortium study, Release 2.0.1 [31]. Recently, our group constructed and validated a P factor model [32] in ABCD from the Child Behavior Checklist (CBCL) parent report [33] using bifactor modeling, and performed additional analyses that support a P factor structural model of psychopathology in this sample [32, 34], see also similar work by [16, 35]. For the present study, we produced resting-state connectomes for 6593 youth who met stringent neuroimaging quality-control standards. We next applied network contingency analysis (NCA) [36–38] to link functional-connectivity patterns to the P factor. NCA uses a count-based statistic to identify network cells (connections linking pairs of networks) where the number of connections linked to a phenotype of interest—in this case the P factor—exceeds the number expected by chance (established through non-parametric permutation tests). We demonstrate that the P factor is associated with widespread altered connectivity patterns prominently implicating networks involved in spontaneous response generation (DMN, VAN) and control networks involved in response control (FPN, DAN, and CO). We further establish that P-factor-related changes affect fine-grained patterns of positive and negative connectivity across connections, and these changes predominantly take an attenuating form: neurotypically positive connections become less positive and neurotypically negative connections become less negative. Finally, we show that these attenuating changes are unlikely to be explained by head motion or household/neighborhood disadvantage [39–42].

## Methods

### Sample and data

The ABCD study is a multisite longitudinal study with 11,875 children between 9 and 10 years of age from 21 sites across the United States. The study conforms to the rules and procedures of each site’s Institutional Review Board, and all participants provided informed consent (parents) or assent (children).

### Data acquisition, fMRI preprocessing, and connectome generation

Imaging protocols were harmonized across sites and scanners. High spatial (2.4 mm isotropic) and temporal resolution (TR = 800 ms) resting-state fMRI was acquired in four separate runs (5 min per run, 20 min total). The entire data pipeline described below was run through automated scripts on the University of Michigan’s high-performance cluster, and is described below, with additional detailed methods automatically generated by fMRIPrep software provided in the Supplement.

Preprocessing was performed using fMRIPrep version 1.5.0 [43]. Full details of the fMRIPrep analysis can be found in Supplemental materials. Briefly, T1-weighted (T1w) and T2-weighted images were run through recon-all using FreeSurfer v6.0.1. T1w images were also spatially normalized nonlinearly to MNI152NLin6Asym space using ANTs 2.2.0. Each functional run was corrected for field-map distortions, rigidly coregistered to the T1, motion corrected, and normalized to standard space. ICA-AROMA was run to generate aggressive noise regressors. Anatomical CompCor was run and the top five principal components of both CSF and white matter were retained. Functional data were transformed to CIFTI space using HCP’s Connectome Workbench. All preprocessed data were visually inspected at two separate stages to ensure only high-quality data was included; after coregistration of the functional data to the structural data and after registration of the functional data to MNI template space.

Connectomes were generated for each functional run using the Gordon 333 parcel atlas [44], augmented with parcels from high-resolution subcortical [45] and cerebellar [46] atlases. Volumes exceeding a framewise displacement threshold of 0.5 mm were marked to be censored. Covariates were regressed out of the time series in a single step, including: linear trend, 24 motion parameters (original translations/rotations + derivatives + quadratics), aCompCorr 5 CSF and 5 white matter components and ICA-AROMA aggressive components, high-pass filtering at 0.008 Hz, and censored volumes. Next, correlation matrices were calculated for each run. Each matrix was then Fisher *r*-to-*z* transformed, and then averaged across runs for each subject yielding their final connectome. A quality-control resting-state functional-connectivity plot is shown in Supplement Fig. S1.

### Constructing a structural model of psychopathology

The general psychopathology factor (P factor) used here is based on the parent-rated CBCL [33], age 6–18 form. A bifactor model was fit to eight CBCL scales, with a general P factor that all scales loaded onto (average-scale loading = 0.69) and two specific factors. This model is described in detail in our previous studies in ABCD [32, 34] and in the Supplement.

### Inclusion/exclusion

There are 11,875 subjects in the ABCD Release 2.0.1 dataset. Screening was initially done using ABCD raw QC to limit to subjects with two or more good runs of resting data, as well as a good T1 and T2 image (QC score, protocol compliance score, and complete all = 1). This resulted in 9580 subjects with two or more runs that entered preprocessing. Each run was subsequently visually inspected for registration and warping quality, and only those subjects who still had two or more good runs were retained (*N* = 8858). After connectome generation, runs were excluded if they had less than 4 min of uncensored data, and next subjects were retained only if they had two or more good runs (*N* = 6595). Finally, subjects who were missing data required for regression modeling (P factor scores or any of the nuisance covariates) were dropped. This left us with *N* = 6593 subjects across 22 sites for the whole-sample NCA analysis. Further information about demographics and psychopathology in this sample are shown in Supplement Tables S1 and S2.

### Network contingency analysis

**Fig. 1:**
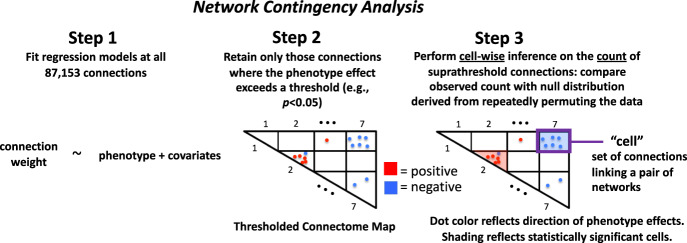
Steps for network contingency analysis (NCA). NCA is a count-based method for quantifying altered connectivity at network cells, i.e., sets of connections linking pairs of large-scale networks. The method assesses, for each cell, whether the count of connections that are significantly related to the phenotye of interest exceeds the number expected by chance (based on non-parametric permutation tests).

NCA is a count-based approach to quantifying altered connectivity (Fig. 1), see our prior work [36, 37] for more details, and see also [47] for a related statistical treatment. In the current application, we applied the approach to “cells”; each cell is the set of connections linking a pair of the 16 networks in the Gordon parcellation (120 total cells). In Step 1 of the analysis, we fit a multiple regression model at each edge of the connectome with edge connectivity weight as the outcome variable and P factor scores as the predictor of interest, while including sex, race, age, age^2^, mean FD, and mean FD^2^ as covariates. In Step 2, we identified all connections in which the P factor effect exceeds (is more significant than) a *p* < 0.05 threshold (“NCA threshold”). In Step 3, we counted the suprathreshold connections separately for each cell, assessing whether this number exceeds the number that would be expected by chance alone. The distribution under chance was generated by non-parametric permutation tests [48]. We randomly shuffled subjects’ edgewise connectivity weights 10,000 times (i.e., subject_*i*_’s edge weights were randomly switched with subject_*j*_’s) and recalculated the count of suprathreshold edges for each cell at each iteration. Permutation *p*-values were then calculated and corrected for multiple comparisons across 120 cells using the false discovery rate (FDR) [49] with alpha set at *p* < 0.05. The procedure of Freedman and Lane [50] was used to account for covariates. In addition, exchangeability blocks were used to account for twin, family, and site structure and were entered into Permutation Analysis of Linear Models (PALM) [51] to produce permutation orderings, as described in detail in the Supplement.

For the main analysis, we set the NCA threshold at *p* < 0.05. We assessed the robustness of our results by repeating the analysis using an average of five thresholds, specifically {0.1, 0.05, 0.01, 0.005, 0.001}, following a procedure based on [47] (see details in the Supplement). Because studies in ABCD found brain changes in relation to household income [40, 42] and neighborhood disadvantage [39, 41], we in addition re-ran the NCA analysis with household income and an index of neighborhood disadvantage (derived from work by Taylor and colleagues [41]) as additional covariates (see details in the Supplement).

### Quadrant analysis of directionality of P effects in relation to the neurotypical connectome

For all the cells identified as modulated by the P factor in the NCA analysis, we next assessed the nature and direction of P-associated changes in connectivity in these cells. In particular, we performed a quadrant analysis [38] assessing how P factor effects relate to positive and negative connectivity in the neurotypical connectome. First, we residualized effects of nuisance covariates from each edge of the connectome (sex, race, age, age^2^, mean FD, and mean FD^2^), and we then computed the mean for each edge. Next, we located all the connections in the NCA-significant cells in one of four quadrants according to directionality of mean connectivity (*x* axis) and directionality of P factor effects (*y* axis). Importantly, the P factor produces attenuating changes if there is a preponderance of connections in quadrant 2 (negative mean connectivity, positive P factor effect) and quadrant 4 (positive mean connectivity, negative P factor effect). Analogously, amplification is indicated by a preponderance of connections in quadrants 1 and 3. We assessed the significance of the observed proportion of edges in the attenuating quadrants with non-parametric permutation tests in which we randomly shuffled the 6593 subjects’ P factor scores 10,000 times and recomputed the proportion of edges in the attenuating quadrants at each iteration. Finally, we additionally performed this quadrant directionality analysis separately at each NCA-significant cell.

### Sensitivity analysis in a low-motion subsample

We assessed the sensitivity of our analysis to artifactual effects of head motion by repeating the NCA analysis and follow-up quadrant analysis in a low-motion set of subjects with mean FD < 0.2 (*N* = 3155). We qualitatively assessed these results for similarity with those from the main sample. In addition, we performed a low-motion difference test, which quantitatively assesses whether NCA results from the low-motion subsample are lower than the results derived from many random subsamples of the data with the same number of subjects (further details are given in the Supplement). A positive test provides evidence that motion is contributing to the observed results.

## Results

### The P factor is associated with statically significant effects across 28 network cells, with prominent effects on connectivity of DMN and control networks

**Fig. 2:**
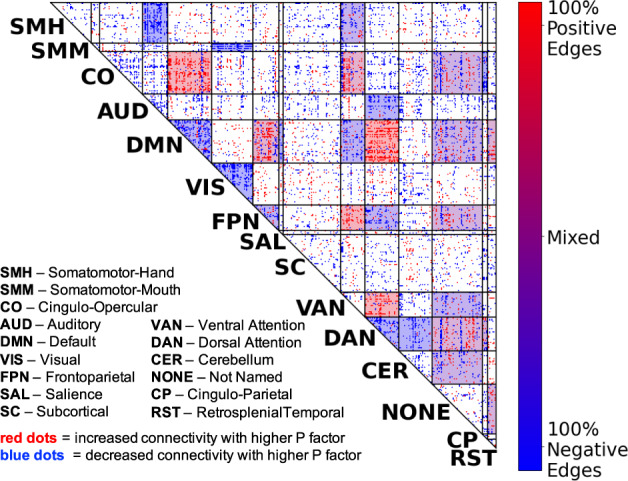
Network-to-network connections exhibiting significant P factor effects. We performed network contingency analysis (NCA) which identifies cells (i.e., sets of connections linking pairs of large-scale networks) where the number of P-factor-related edges exceeds the number expected by chance. A total of 28 cells exhibited significant P factor effects (FDR < 0.05; shaded in the figure). P factor effects were prominent in default network as well in control networks (frontoparietal, dorsal attention, and cingulo-opercular). Other networks prominently implicated were ventral attention and “None” (i.e., no label assigned in the Gordon parcellation).

**Fig. 3:**
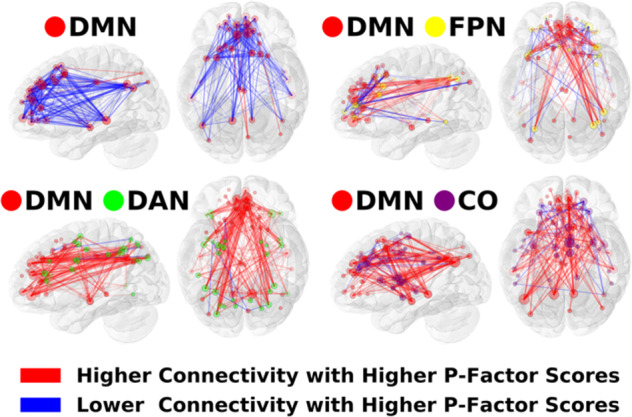
Default mode network connections associated with the P factor. The results from our network analysis showed that the P factor is associated with hypoconnectivity within DMN and hyperconnectivity between DMN and three control networks, FPN, DAN, and CO. DMN = default mode network, FPN = frontoparietal network, DAN = dorsal attention network, CO = cingulo-opercular network.

NCA analysis revealed statistically significant effects of the P factor at 28 network cells (FDR-corrected *p*_PERMUTATION_ < 0.05), see Fig. 2 and Supplement Table S3. P factor effects were prominent within DMN and in DMN’s connections with three control networks, FPN, DAN, and CO. These altered connectivity patterns are shown as 3D brain space visualizations in Fig. 3, which highlights that higher P factor scores are associated with reduced connectivity within DMN and increased connectivity between DMN and control networks. P-factor-related connectivity changes also prominently implicated VAN, including its connections with DMN, FPN, DAN, and CO. Additionally, P factor effects were notable in the “None” network (i.e., no label assigned in the Gordon parcellation), which exhibited altered connectivity with DMN, FPN, DAN, and CO. Brain space visualizations of altered VAN and None connectivity are shown in Supplement Figs. S2 and S3. Additionally, we repeated the preceding NCA analysis using an average of five thresholds (see “Methods” section Network contingency analysis), and the results were highly similar (see Supplement Fig. S4). Also, we repeated the preceding NCA analysis adding household income and an index of neighborhood disadvantage as covariates. Once again, the results were highly similar, with 25 significant cells versus 28 in the original analysis (see Supplement Fig. S5). Finally, we repeated quadrant analyses (see below) for both of the preceding analyses (i.e., weighted average of five thresholds and household income/neighborhood disadvantage as covariates). In both cases, percentage of attenuating connections was nearly identical (67% and 68%, respectively) and their *p*-values remained highly statistically significant (*p*_PERMUTATION_ < 0.0001).

### Within these 28 P-factor-affected cells, P factor effects were primarily attenuating

**Fig. 4:**
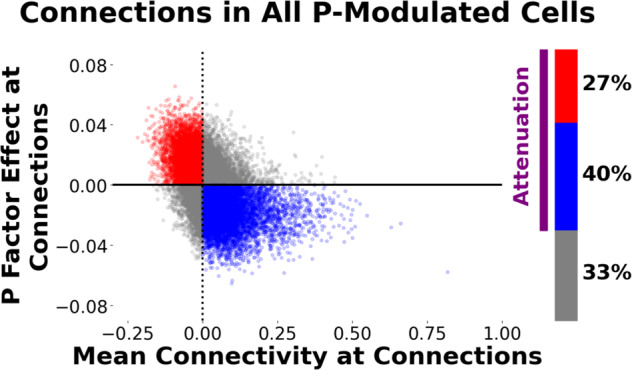
Quadrant analysis demonstrating attenuating effects of the P factor on neurotypical functional-connectivity patterns. In the main NCA analysis, we identified 28 cells (i.e., sets of connections linking pairs of large-scale networks) that were significantly associated with the P factor. In a follow-up quadrant analysis, we placed these connections in one of four quadrants according to directionality of mean connectivity (*x* axis) and directionality of P factor effects (*y* axis); in the figure, each dot represents one connection. We found 67% of connections reside in quadrants corresponding to attenuating effects, i.e., quadrant 2 shown in red (negative mean connectivity, positive P factor effect) and quadrant 4 shown in blue (positive mean connectivity, negative P factor effect). Non-parametric permutation tests showed that this elevated proportion of attenuating P factor effects was highly unlikely to have arisen by chance *(p*_PERMUTATION_ < 0.0002).

We used quadrant analysis to assess the directionality of P factor effects within the 28 cells found to be significant in NCA analysis. As shown in Fig. 4, we found that 67% of connections resided in quadrant 2 (negative neurotypical connectivity, positive P factor effect) and quadrant 4 (positive neurotypical connectivity, negative P factor effect)—a pattern that reflects attenuation of neurotypical connectivity by the P factor. Permutation testing revealed that this elevated percentage of attenuating P factor effects is highly unlikely to have arisen by chance (*p*_PERMUTATION_ < 0.0002). We additionally conducted quadrant analysis on each of the 28 cells separately. We found a statistically significant proportion of attenuation (i.e., elevated counts of connections in quadrants 2 and 4) in 18 out of the 28 cells considered individually (*p*_PERMUTATION_’s < 0.05).

### The preceding results remained significant in a low-motion subsample

We repeated the preceding NCA analysis and quadrant analysis in a low-motion subsample of subjects with mean FD < 0.2 (*N* = 3155). We found that 25 out of 28 cells identified as FDR < 0.05 significant in the full sample remained FDR < 0.05 significant in the low-motion subsample, see Supplement Fig. S6. In addition, we found that 67% of the connections within NCA-significant cells exhibited the attenuating pattern (the same percentage as in the full sample), see Supplement Fig. S7. The associated *p*-value from permutation testing was *p*_PERMUTATION_ < 0.0001 (observed proportion of attenuating effects was larger than all values in the permutation distribution). Finally, low-motion difference tests applied to NCA cell counts were not significant for all 120 cells (mean *p* = 0.79), providing additional evidence that our NCA analysis results are not due to head motion.

## Discussion

This study investigated brain-wide connectomic alterations associated with the general factor of psychopathology (“P factor”), a factor representing broad expression of prevalent psychiatric symptoms, in 6593 9- and 10-year olds in the ABCD multisite sample. The results support three major findings. First, by combining large sample size, rigorous non-parametric network methods, and explicit tests of sensitivity to motion, we provide particularly compelling evidence that the P factor is associated with widespread alterations in connectivity across the brain’s intrinsic functional architecture. Second, we found especially prominent alterations in networks involved in generation of spontaneous responses (DMN, VAN) and control networks involved in response control (FPN, DAN, and CO). Third, we demonstrated that within the affected cells (i.e., sets of connections linking pairs of large-scale networks), P factor effects are primarily attenuating: they disproportionately make neurotypically positive connections less positive and neurotypically negative connections less negative. Overall, these results advance understanding of network abnormalities associated with broad liabilities for psychopathology during the transition to adolescence, a key developmental window in which many serious psychopathologies first emerge.

Recent theoretical discussions [3, 13, 34] conceptualize the P factor in terms of a dysregulation model in which there is heightened generation of spontaneous impulses (both negative fear/distress emotions as well as reward-seeking impulses) and reduced executive regulation of these impulses. This model is suggestively reflected in our findings, where we found altered connectivity in networks associated with spontaneous thought (DMN) [52, 53] and bottom-up attention (VAN) [54], as well as altered connectivity in control networks (FPN, CO, DAN) involved in response control [14, 55]. Notably, other recent studies of the P factor examining functional connectivity also found alterations in networks involved in bottom-up processing, including DMN [16], visual network [15], and somatomotor network [18]. Altered functional connectivity in core control networks (i.e., FPN and DAN), however, appears to be a relatively new finding reported here, and our results thus provide firmer grounding for the dysregulation model of the P factor. Of note, previous studies in adolescents and young adults [56, 57] found P-factor-related alternations in cerebellum and interpreted these results in terms of cerebellar contributions to cognitive control (though studies of adults failed to replicate this finding [58]). We here observed alterations in connectivity between cerebellum and DAN, adding to evidence linking cerebellar alterations to the P factor, especially in younger individuals.

We additionally found that within P factor-modulated network cells, the P factor’s effects were predominantly attenuating: the P factor effect disproportionately makes neurotypically positive connections less positive and neurotypically negative connections less negative, thus effectively shrinking connection weights towards zero. Moreover, we demonstrated these results were unlikely to be due to head motion, since they remained, and indeed tended to be qualitatively stronger, in a low-motion subsample (and tests assessing whether the results from a low-motion sample differed from similarly sized random subsamples were not significant). One explanation of the observed pattern of distributed connectivity attenuation associated with the P factor is based on neurodevelopment. The functional connectome undergoes substantial maturation during youth [22–24]. During the course of this neurodevelopmental sequence, individual connections gradually differentiate and exhibit fine-grained patterns of variation in connectivity strength [23]. These changes occur through complex and choreographed processes of integration [59], in which connectivity between nodes is enhanced, and segregation, wherein nodes become increasingly anti-correlated [29, 60]. If this neurodevelopmental sequence is perturbed or disrupted, strength of integration and segregation would be diminished, thus producing a relatively attenuated pattern of connectivity in the affected connectome relative to a neurotypical connectome. Future work should attempt to better understand the neurodevelopmental origins and consequences of the pattern of widespread attenuation of connectivity identified in this study as linked to the P factor. Of note, upcoming waves of data from the ABCD longitudinal study could be particularly illuminating, as they can shed light on whether this pattern of attenuated connectivity worsens, improves, or holds steady with age, and whether at the single subject level, attenuated connectivity co-matures across adolescence with levels of the P factor.

Our results are relevant to interpreting previous functional-connectivity studies in psychiatric imaging that mostly used case–control designs [61]. These studies aimed to characterize brain network abnormalities associated with individual disorders, where these abnormalities were assumed to reflect disorder-specific pathophysiology. Accumulated results, however, suggest observed network alterations often lack specificity. For example, two prominent network motifs we observed in the present study, reduced connectivity within DMN and altered DMN/control networks connectivity, have elsewhere been demonstrated in a number of individual psychiatric disorders, including hypoconnectivity of DMN in autism [62], schizophrenia [63], and ADHD [36, 64, 65], and reduced DMN/control network anti-correlation in schizophrenia [66–68], bipolar disorder [69], and ADHD [36, 65]. The present study potentially explains this lack of specificity by linking motifs such as these instead to the P factor. The P factor represents broad expression of diverse forms of psychopathology, and thus P-linked connectomic motifs would be expected to show up nonspecifically across diverse case–control disorder comparisons.

This study has several limitations. First, construction of a P factor from symptom-scale data requires making certain modeling choices (e.g., bifactor versus higher-order factor modeling, item- or scale-level inputs, etc.). This concern is potentially mitigated, however, by findings from our recent report [32] using the same ABCD data used here, in which we showed that across 14 such modeling choices, resulting P factor variables were highly consistent (*r*’s > 0.90). Second, this study was conducted in 9- and 10-year-old youth, many of whom had relatively low levels of psychiatric symptomatology (Table S2). It is expected that subjects’ psychopathology load will rise during the course of adolescence [26], and it is possible that brain/behavior relationships could correspondingly become stronger or otherwise change, a hypothesis that can be directly tested in future waves of longitudinal ABCD data. Third, the NCA method we used relies on a prespecified parcellation of the brain into networks in order to perform inference on connections linking pairs of networks (note: we used the parcellation by Gordon and colleagues [44]). It is possible that future work may identify parcellations that perform still better for NCA-type inference. Finally, this study exclusively examined brain/behavior relationships with resting-state functional connectomes. Recent work links the P factor to structural alterations [20, 56, 70] (gray matter reductions) and white matter tract changes [56, 71], and concurrent investigation of multiple modalities (multi-modal fusion methods) could yield a more complete understanding of the brain basis of the P factor.

In sum, we found that during emerging adolescence, the P factor is associated with distributed attenuation of connectivity in networks involved in spontaneous response generation and networks involved in cognitive control, critical elements of the brain’s intrinsic functional architecture. These findings set the stage for future studies in the ABCD sample that leverage longitudinal waves of data to trace the psychological and neural progression of the P factor during a critical window of vulnerability to mental illness that spans adolescence to young adulthood.

## Supplementary information

Supplemental Methods and Results.

Supplemental FMRIPrep Methods.

## Data Availability

The ABCD data used in this report came from ABCD Release 2.1, NDA Study 721, 10.15154/1504041, which can be found at https://nda.nih.gov/study.html?id=721. The specific NDA study associated with this report is NDA Study 1365, 10.15154/1523387.

## Code availability

Code for running this analysis can be found at https://github.com/SripadaLab/ABCD_Resting_PFactor_NCA
