# Supplementary material for: Widespread attenuating changes in brain connectivity associated with the general factor of psychopathology in 9- and 10-year olds
Source: Transl Psychiatry. 2021 Nov 9;11:575. doi: 10.1038/s41398-021-01708-w (PMC8578613; doi:10.1038/s41398-021-01708-w)
Supplement: Supplementary file 1 — Supplemental Methods and Results. [file 41398_2021_1708_MOESM1_ESM.docx]

**Supplement**

**Supplementary Methods**

**1. Connectome Generation and Quality Control-Resting State Functional Connectivity Plot**


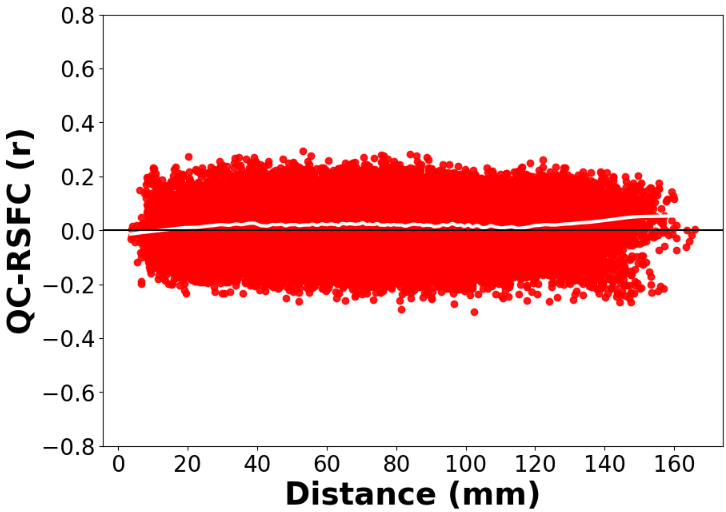


***Figure S1: Quality Control-Resting State Functional Connectivity Plot***

We used multiple procedures to control the effect of head motion on brain-behavior relationships, which are listed in §2.2 in the Main Manuscript. To assess the effectiveness of these procedures, we produced a quality control resting state functional connectivity (QC-RSFC) plot (1,2). This plot shows the relationship between mean framewise displacement and connectivity for edges binned by distance. Motion effects produce a sloped line (distance-dependent artifact), while a flat line is indicative of minimal motion-related effects. The RSFC-QC plot for our ABCD resting state data showed a flat line, providing additional evidence that our stringent motion correction strategies were effective.

**2. P Factor Modeling**

A general P-factor with two orthogonal specific factors were modeled using the parent-rated Child Behavior Checklist (CBCL), age 6 to 18 form (3). We fit a bifactor model to the eight CBCL scales (Withdrawn, Somatic Complaints, Anxious/Depressed, Social Problems, Thought Problems, Attention Problems, Delinquent Behavior, and Aggressive Behavior). In this model there was a general P factor that all scales loaded onto (average scale loading on P = .69), and internalizing and externalizing specific factors (average scale loading = .43). This model fit well based on conventional fit thresholds (χ^2^ = 747.73, df = 16, *p* < .001; RMSEA = .062; CFI = .985; TLI = .974; SRMR = .015) and was chosen for its good model fit and theoretical interpretability. Importantly, we elsewhere demonstrate that across a variety of alternative specifications of hierarchical models of psychopathology in ABCD, the resulting P factors that emerge yield a highly similar rank ordering of subjects (rs>0.90) (4), rendering decisions about which specific modeling strategy to adopt less consequential.

**3. Permutation Framework**

Network contingency analysis (NCA) assesses whether the observed count of suprathreshold edges in a cell is higher than the count that is expected by chance. Schematically, the procedure is as follows (note: the actual procedure is more complicated owing to the presence of covariates, as outlined in the subsequent paragraph): Distributions under chance of counts of suprathreshold edges were generated by randomly shuffling the subjects’ edge-wise connectivity weights 10,000 times (i.e., subject_i_’s edge weights are randomly switched with subject_j_’s). At each iteration, we performed the edge-wise regressions described in step 1 of NCA (see Methods §2.5 of the main manuscript), and then recalculated the count of suprathreshold edges. The observed count of suprathreshold edges was located in this null distribution in terms of rank, and a *p*-value was obtained by dividing this rank value by 10,000, corresponding to a one-sided test of the hypothesis that the observed count of suprathreshold edges exceeds that expected by chance.

Since the P factor regression models fit at each iteration of the permutation procedure included covariates, the procedure of Freedman and Lane (5) was followed. Since subjects were nested within families and site, exchangeability blocks (6) were generated and entered into FSL’s Permutation Analysis of Linear Models (PALM) tool (<https://fsl.fmrib.ox.ac.uk/fsl/fslwiki/PALM>) to generate 10,000 permutation orderings. At each iteration, we first fit the outcome variables (i.e., edge connectivity weights) to a model containing only nuisance predictors. We calculate the residuals from this model, permute them within exchangeability blocks, then add the unpermuted estimated effect of nuisance predictors back to the permuted residuals. This new outcome variable, made up of permuted residuals and unpermuted nuisance effects, is then fit to the regression models to generate a test statistic (count of suprathreshold edges in this case). This sequence is then repeated 10,000 times in total to generate the permutation null distribution of the test statistic.

**4. Network Contingency Analysis with Average of Multiple Thresholds**

We performed the main NCA analysis with a *p* < 0.05 threshold. We assessed the robustness of our results by repeating the analysis using an average of five thresholds, specifically {0.1, 0.05, 0.01, 0.005, 0.001}. To facilitate aggregation, we first standardized each count statistic according the formula from Donoho and Jin (7) as follows:

$$W\left( \alpha\right)=\sqrt{n}\frac{\left( Fraction Significant at \alpha\right)-\alpha}{\sqrt{\alpha\left( 1-\alpha\right)}}$$

Our test statistic *T* is the average of *W*(α) taken over {0.1, 0.05, 0.01, 0.005, 0.001}. For each cell, we calculate the observed *T* and obtain a *p*-value by comparing it to the distribution of *T* obtained from permuted data, analogous to our approach for the main NCA analysis.

**5. Household Income and Neighborhood Disadvantage**

Household Income covered all sources of income for family members, including wages, benefits, child support payments, and others. It was assessed in bins as follows: 1 <5,000, 2 5,000 - 11,999, 3 12,000 - 15,999, 4 16,000 - 24,999, 5 25,000 - 34,999, 6 35,000 - 49,999, 7 50,000 - 74,999, 8 75,000 - 99,999, 9 100,000 - 199,999, 10 More than 200,000, and we assigned each subject the natural log of the midpoint for their bin. Highest Parental Educational was the highest educational achievement by either parent or caregiver. Neighborhood Disadvantage was constructed according to the procedure of Taylor et al. (8). In brief, participant’s primary home address was used to generate Area Deprivation Index (ADI) values, which were factor analyzed and used to create an aggregate measure. Higher scores on the factor indicate greater neighborhood disadvantage including higher percent of families living in poverty, increased unemployment, and lower levels of educational attainment at the neighborhood level, see (8) for details.

**6. Low Motion Difference Test**

To further assess whether motion is contributing to our NCA results, we performed a “low motion difference test” based on resampling. First, we created a low motion subsample of the data with *n*=3,155, and we performed NCA in this subsample. Next, we generated 10,000 same-sized random subsamples and performed NCA in each. We assigned a *p*-value for each cell by locating the count of suprathreshold edges in the low motion sample in that cell’s associated resampling distribution. We declared the result significant if the one-tailed *p*-value was below 0.05, indicating that the cell count for the true low motion subsample is lower than those from random subsamples of the same size.

**Supplementary Results**

**1. Additional 3D Brain Space Visualizations of Network Contingency Analysis Results**


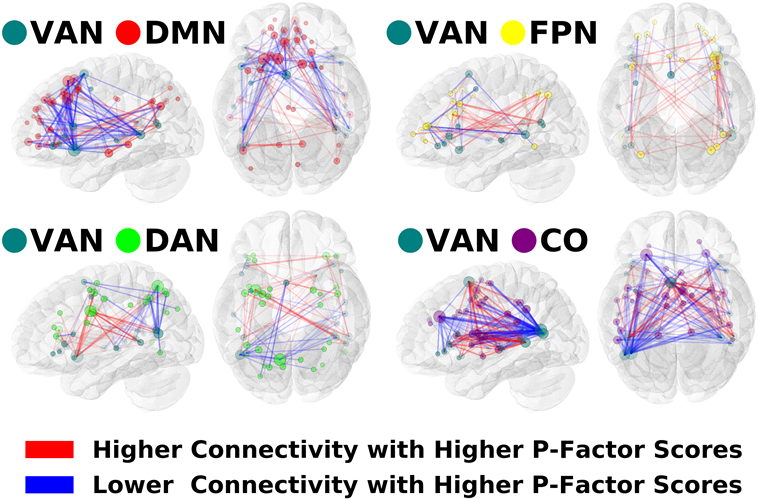


***Figure S2: Ventral Attention Network Connections Associated with the P Factor.*** *Results from our network analysis showed that the P factor is associated with altered connectivity between VAN and DMN, FPN, DAN, CO.*


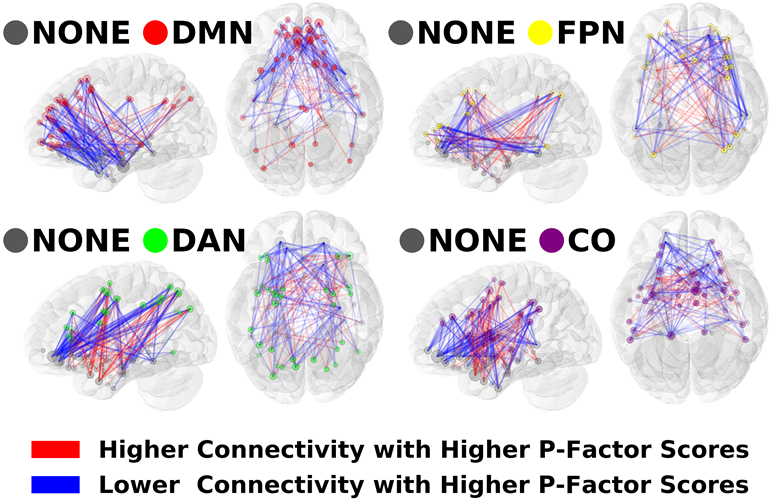


***Figure S3: None Network Connections Associated with the P Factor.*** *Results from our network analysis showed that the P factor is associated with altered connectivity between the None Network (no label assigned in the Gordon Parcellation) and DMN, FPN, DAN, CO.*

**2. Results for Network Contingency Analysis Using Weighted Average of Thresholds**

***Figure S4: Network Contingency Analysis of P Factor Effects with Weighted Average of Thresholds.*** *We performed a network contingency analysis (NCA) for the P factor using a weighted average of five thresholds. Results were highly similar the main analysis that used a p < 0.05 threshold, confirming the robustness of results to selection across thresholds.*

***Figure S5: Network Contingency Analysis of P Factor Effects with Additional Covariates for Household Income and Neighborhood Disadvantage.*** *We performed a network contingency analysis (NCA) for the P factor with additional covariates for household income and an index of neighborhood disadvantage. Results were highly similar the main analysis that used a p < 0.05 threshold.*

**3. Results for Network Contingency Analysis in Low Motion Subsample**

***Figure S6: Network Contingency Analysis of P Factor Effects with Low Motion Subsample.*** *We performed a network contingency analysis (NCA) for the P factor in a subsample with low head motion (FD < 0.02). Results were highly similar the main analysis, and indeed qualitatively appeared to be somewhat stronger (43 significant cells compared to 28 significant cells in the main analysis).*

**
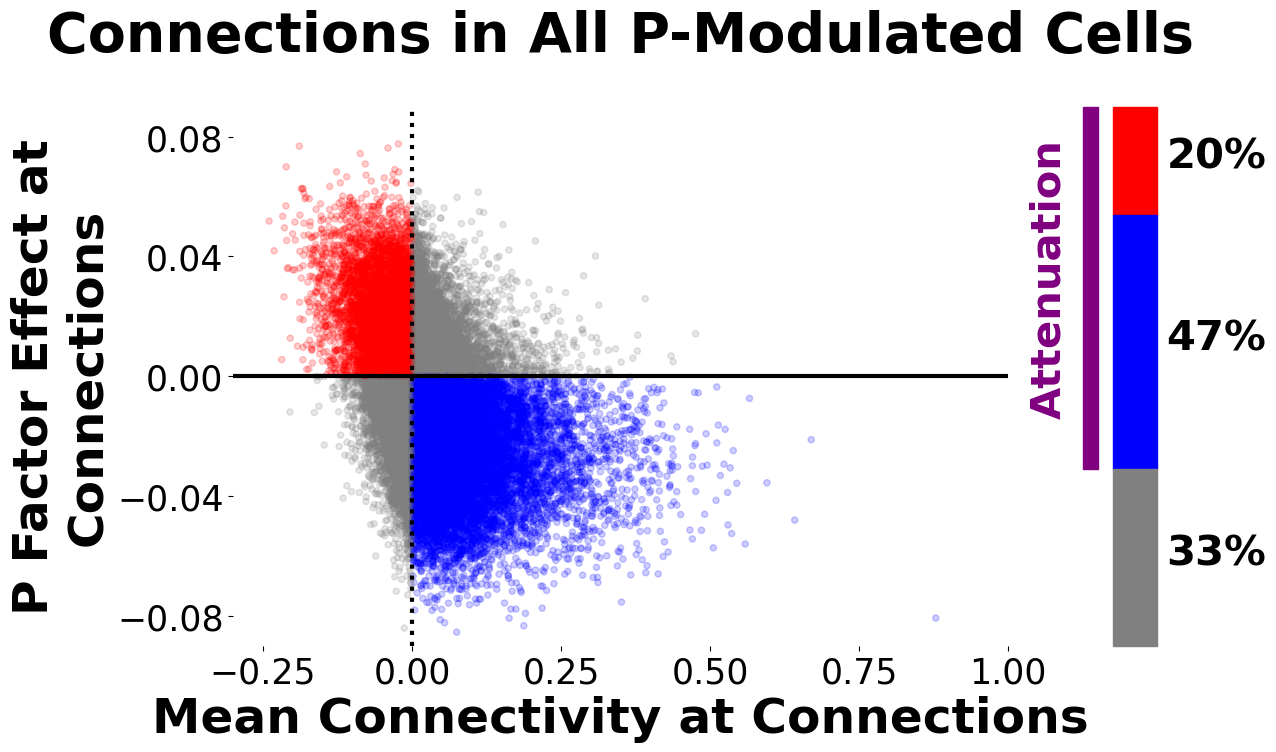
**

**Figure S7: *Quadrant Analysis in Low Motion Subsample Demonstrating Attenuating Effects of the P Factor on Neurotypical Functional Connectivity Patterns.*** *We performed quadrant analysis in which we placed all connections in NCA-significant cells in one of four quadrants according to directionality of mean connectivity (x axis) and directionality of P factor effects (y axis); in the figure, each dot represents one connection. We found 67% of connections reside in quadrants corresponding to attenuating effects, i.e., quadrant 2 shown in red (positive mean connectivity, negative P factor effect) and quadrant 4 shown in blue (negative mean connectivity, positive P factor effect). Non-parametric permutation tests showed that this elevated proportion of attenuating P factor effects was highly unlikely to have arisen by chance (p_PERMUTATION_<0.0001), observed proportion is larger than all values in the permutation distribution.*

|  | Included | Excluded |
| --- | --- | --- |
| N | 6593 | 5282 |
| Age (mean (s.d.)) | 9.85 (0.62) | 9.96 (0.62) |
| Female (%) | 3328 (50.5) | 2353 (44.5) |
| Race Ethnicity (%) |  |  |
| White | 3846 (58.3) | 2332 (44.1) |
| Black | 808 (12.3) | 972 (18.4) |
| Hispanic | 1203 (18.2) | 1206 (22.8) |
| Asian | 118 (1.8) | 155 (2.9) |
| Other | 618 (9.4) | 617 (11.7) |
| No answer | -- | -- |
| Highest Parental Education (%) |  |  |
| < HS Diploma | 230 (3.5) | 362 (6.9) |
| Bachelor | 1793 (27.2) | 1221 (23.1) |
| HS Diploma/GED | 488 (7.4) | 643 (12.2) |
| Post Graduate Degree | 2442 (37.0) | 1601 (30.3) |
| Some College | 1637 (24.8) | 1441 (27.3) |
| No answer | 3 (0.05) | 14 (0.3) |
| Household Marital Status – Married (%) | 4732 (71.8) | 3258 (61.7) |
| Household Income (%) |  |  |
| <50K | 1546 (23.4) | 1676 (31.7) |
| >=100k | 2739 (34.6) | 1826 (34.6) |
| >=50k & <100K | 1816 (23.7) | 1254 (23.7) |
| No answer | 492 (7.5) | 526 (10.0) |

***Table S1: Demographic Characteristics of Included Versus Excluded Subjects***

|  | Included | Excluded |
| --- | --- | --- |
| KSADS Diagnoses (count (%)) |  |  |
| ADHD | 1228 (18.6) | 1323 (25.0) |
| Anxiety | 2358 (35.8) | 1963 (37.2) |
| Bipolar | 389 (5.9) | 415 (7.9) |
| Depression | 376 (5.7) | 365 (6.9) |
| Developmental | 1636 (24.8) | 1611 (30.5) |
| Eating | 713 (10.8) | 494 (9.4) |
| OCD | 658 (10.0) | 602 (11.4) |
| Oppositional Defiant/Conduct | 906 (13.7) | 892 (16.9) |
| PTSD | 264 (4.0) | 286 (5.4) |
| Psychosis | 165 (2.5) | 150 (2.8) |
| Substance | 9 (0.1) | 18 (0.3) |
| Suicide/Self-Injury | 609 (9.2) | 543 (10.3) |
| CBCL Raw Scores (mean (s.d.)) |  |  |
| Aggressive | 3.0 (4.2) | 3.5 (4.6) |
| Anxious/Dep | 2.5 (3.1) | 2.5 (3.0) |
| Attention | 2.7 (3.3) | 3.3 (3.7) |
| Rule Breaking | 1.1 (1.7) | 1.3 (2.0) |
| Social | 1.5 (2.2) | 1.8 (2.4) |
| Somatic | 1.5 (1.9) | 1.5 (2.0) |
| Thought | 1.6 (2.1) | 1.7 (2.3) |
| With/Dep | 1.0 (1.7) | 1.1 (1.8) |
| Total Problems | 17.1 (17.3) | 19.5 (18.7) |
|  |  |  |

***Table S2: Clinical Information of Included Versus Excluded Subjects***

| \| **Cell** \| \| \| **Raw Permutation P** \| **FDR-**  **corrected P** \| \| --- \| --- \| --- \| --- \| --- \| \| DMN \| - \| DMN \| <0.0001 \| 0.0024 \| \| DMN \| - \| FPN \| <0.0001 \| 0.0024 \| \| DMN \| - \| DAN \| <0.0001 \| 0.0024 \| \| DAN \| - \| NONE \| <0.0001 \| 0.0024 \| \| CO \| - \| VAN \| 0.0001 \| 0.0024 \| \| SMM \| - \| VIS \| 0.0004 \| 0.0060 \| \| FPN \| - \| VAN \| 0.0004 \| 0.0060 \| \| DMN \| - \| NONE \| 0.0005 \| 0.0060 \| \| FPN \| - \| NONE \| 0.0005 \| 0.0060 \| \| DAN \| - \| DAN \| 0.0005 \| 0.0060 \| \| CO \| - \| DMN \| 0.0006 \| 0.0065 \| \| SMH \| - \| AUD \| 0.0007 \| 0.0070 \| \| FPN \| - \| DAN \| 0.0008 \| 0.0074 \| \| FPN \| - \| FPN \| 0.001 \| 0.0086 \| \| CO \| - \| NONE \| 0.0012 \| 0.0090 \| \| VIS \| - \| VIS \| 0.0012 \| 0.0090 \| \| NONE \| - \| RST \| 0.0017 \| 0.0120 \| \| VAN \| - \| DAN \| 0.0018 \| 0.0120 \| \| DMN \| - \| VAN \| 0.002 \| 0.0126 \| \| VAN \| - \| NONE \| 0.0021 \| 0.0126 \| \| DAN \| - \| CP \| 0.0024 \| 0.0137 \| \| CER \| - \| NONE \| 0.003 \| 0.0164 \| \| DMN \| - \| SAL \| 0.0043 \| 0.0224 \| \| CO \| - \| CP \| 0.0048 \| 0.0240 \| \| SMH \| - \| VAN \| 0.0074 \| 0.0355 \| \| AUD \| - \| DAN \| 0.008 \| 0.0369 \| \| DMN \| - \| RST \| 0.0101 \| 0.0449 \| \| DAN \| - \| CER \| 0.0105 \| 0.0450 \| |  |
| --- | --- | --- | --- | --- | --- | --- | --- | --- | --- | --- | --- | --- | --- | --- | --- | --- | --- | --- | --- | --- | --- | --- | --- | --- | --- | --- | --- | --- | --- | --- | --- | --- | --- | --- | --- | --- | --- | --- | --- | --- | --- | --- | --- | --- | --- | --- | --- | --- | --- | --- | --- | --- | --- | --- | --- | --- | --- | --- | --- | --- | --- | --- | --- | --- | --- | --- | --- | --- | --- | --- | --- | --- | --- | --- | --- | --- | --- | --- | --- | --- | --- | --- | --- | --- | --- | --- | --- | --- | --- | --- | --- | --- | --- | --- | --- | --- | --- | --- | --- | --- | --- | --- | --- | --- | --- | --- | --- | --- | --- | --- | --- | --- | --- | --- | --- | --- | --- | --- | --- | --- | --- | --- | --- | --- | --- | --- | --- | --- | --- | --- | --- | --- | --- | --- | --- | --- | --- | --- | --- | --- | --- | --- | --- | --- | --- | --- |

***Table S3: Significant Cells in Main Network Contingency Analysis.*** *Network contingency analysis identified 28 cells, listed above, in which the number of suprathreshold connections exceeds what would be expected by chance. Permutation p-value of “<0.0001” means the observed number of suprathreshold edges exceeds all values in the permutation distribution. SMH – Somatomotor-Hand, SMM – Somatomotor-Mouth, CO – Cingulo-Opercular, AUD – Auditory, DMN – Default, VIS – Visual, FPN – Frontoparietal, SAL – Salience, SC – Subcortical, VAN – Ventral Attention, DAN – Dorsal Attention, CER – Cerebellum, NONE – Not Named, CP – Cingulo-Parietal, RST – RetrosplenialTemporal.*

**Supplement References**

1. Power JD, Mitra A, Laumann TO, Snyder AZ, Schlaggar BL, Petersen SE. Methods to detect, characterize, and remove motion artifact in resting state fMRI. Neuroimage. 2014 Jan 1;84:320–41.

2. Power JD, Schlaggar BL, Petersen SE. Recent progress and outstanding issues in motion correction in resting state fMRI. Neuroimage. 2015 Jan 15;105:536–51.

3. Achenbach TM, Ruffle TM, others. The Child Behavior Checklist and related forms for assessing behavioral/emotional problems and competencies. Pediatrics in review. 2000;21(8):265–71.

4. Clark DA, Hicks BM, Angstadt M, Rutherford S, Taxali A, Hyde LW, et al. The General Factor of Psychopathology in the Adolescent Brain Cognitive Development (ABCD) Study: A Comparison of Alternative Modeling Approaches. Clinical Psychological Science. 2021;

5. Freedman D, Lane D. A Nonstochastic Interpretation of Reported Significance Levels. Journal of Business & Economic Statistics. 1983;1(4):292–8.

6. Winkler AM, Ridgway GR, Webster MA, Smith SM, Nichols TE. Permutation inference for the general linear model. Neuroimage. 2014;92:381–97.

7. Donoho D, Jin J. Higher criticism for detecting sparse heterogeneous mixtures. The Annals of Statistics. 2004;32(3):962–94.

8. Taylor RL, Cooper SR, Jackson JJ, Barch DM. Assessment of neighborhood poverty, cognitive function, and prefrontal and hippocampal volumes in children. JAMA network open. 2020;3(11):e2023774–e2023774.
